# The cleaner, the greener? Product sustainability assessment of the biomimetic façade paint Lotusan^®^ in comparison to the conventional façade paint Jumbosil^®^

**DOI:** 10.3762/bjnano.7.200

**Published:** 2016-12-29

**Authors:** Florian Antony, Rainer Grießhammer, Thomas Speck, Olga Speck

**Affiliations:** 1 Plant Biomechanics Group, Botanic Garden, Faculty of Biology, University of Freiburg, 79104 Freiburg, Germany; 2 Öko-Institut e.V., Institute for Applied Ecology, 79017 Freiburg, Germany; 3 Competence Network Biomimetics, Germany; 4 Freiburg Materials Research Center (FMF), 79104 Freiburg, Germany; 5 Freiburg Centre for Interactive Materials and Bioinspired Technologies (FIT), 79110 Freiburg, Germany

**Keywords:** biomimetic promise, life-cycle assessment (LCA), Lotus-Effect^®^ technology, Lotusan^®^, product sustainability assessment (PROSA)

## Abstract

**Background:** The debate on the question whether biomimetics has a specific potential to contribute to sustainability is discussed among scientists, business leaders, politicians and those responsible for project funding. The objective of this paper is to contribute to this controversial debate by presenting the sustainability assessment of one of the most well-known and most successful biomimetic products: the façade paint Lotusan^®^.

**Results:** As a first step it has been examined and verified that the façade paint Lotusan^®^ is correctly defined as a biomimetic product. Secondly, Lotusan^®^ has been assessed and compared to a conventional façade paint within the course of a detailed product sustainability assessment (PROSA). For purposes of comparison, the façade paint Jumbosil^®^ was chosen as reference for a conventional paint available on the market. The benefit analysis showed that both paints fulfil equally well the requirements of functional utility. With respect to the symbolic utility, Lotusan^®^ has a particular added aesthetic value by the preservation of the optical quality over the life cycle. Within the social analysis no substantial differences between the two paints could be found regarding the handling and disposal of the final products. Regarding the life-cycle cost, Lotusan^®^ is the more expensive product. However, the higher investment cost for a Lotusan^®^-based façade painting are more than compensated by the longer life time, resulting in both reduced overall material demand and lower labour cost. In terms of the life-cycle impact assessment, it can be ascertained that substantial differences between the paints arise from the respective service life, which are presented in terms of four scenario analyses.

**Conclusion:** In summary, the biomimetic façade paint Lotusan^®^ has been identified as a cost-effective and at the same time resource-saving product. Based on the underlying data and assumptions it could be demonstrated that Lotusan^®^-based façade paintings have a comparatively low overall impact on the environment. Summarizing our results, it can be emphasized that Lotusan^®^ is the more favourable product compared to Jumbosil^®^ according to sustainability aspects.

## Introduction

In-depth analyses of functions found in biology and the systematic transfer of the respective operating principles into technical applications is the essential aim of biomimetics [1–2]. Bringing together the competencies of experts from different scientific disciplines, biomimetics has been successfully established as an independent scientific discipline of continuously increasing visibility [3–4]. Especially in the context of architecture and building technologies a lot of innovative developments were derived from biological examples over the last years [5–7]. The analysis of plant surfaces as contribution to the systematics of plants lead to the discovery of the operating principle of the self-cleaning effect of plant surfaces. This was brought into the construction market as biomimetic self-cleaning façade paint [8]. The study of morphology and anatomy, mechanics and functional principles in biology has the potential to stimulate architects and engineers to new building solutions in architectural design and technical implementation such as the adaptive façade shading system flectofin^®^ [6,9] or the bone-like ceiling of a lecture hall at the University of Freiburg [10–11].

The increasingly systematic research approach of biomimetics, aiming to find the most promising examples from biology for the development of technical innovations, has been accompanied by a debate about the specific potential of biomimetic solutions to contribute to sustainability [12–18]. Gebeshuber et al. also argue in this direction by envisaging possible scenarios of future development by mimicking biology and by promising to overcome some of the major global challenges as indicated by the so-called Millennium Project [19]. More recently and from the perspective of a higher abstraction level of learning from nature, the opportunities of mimicking ecosystem services for regenerative urban design have been analysed [20].

In principle, there are different interpretations of sustainability, but none of those is based on an absolute concept, but on comparisons and process orientation. From the point of view of the authors, sustainability has to be understood as a normative term and refers to a form of economic activity and lifestyle that is based on moral commitments to future generations.

Biomimetic products are often attributed to have an intrinsic potential to contribute to a more sustainable technology, with reference made to the inspiratory flow from living nature [16]. By introducing the term of the so-called “biomimetic promise” Arnim von Gleich characterised the discussion on sustainability in biomimetics. In addition, von Gleich also referred to requirements on the validity and limitations of the biomimetic promise [12]. Also J.F. Vincent expressed his conviction that biomimetics has the potential to contribute to a more sustainable future, while at the same time he warned that it is not sufficient to translate the lessons of nature into the present technology in any event [21]. As von Gleich stated before, the implementation of the biomimetic promise can only be regarded on a case-by-case basis [12,22–23].

From the perspective of scientific theory it might be a normative claim rather than a descriptive one that biomimetic innovations ought to deliver sustainable solutions [16–18]. This leads to the question as to whether bio-inspired innovations also have a specific quality in terms of sustainability [18]. Figuratively speaking, this means that sustainability may be a by-product of knowledge transfer from biology to technology. A much safer approach is the definition of sustainability as an initial objective and its explicit knowledge transfer in the course of the development of bio-inspired and biomimetic innovations [17–18].

However, Raibeck et al. point out that statements touting the benefits of biologically inspired sustainable engineering appear in the literature but also that limited scientific data exist in order to substantiate such statements [24]. Within their article they present a life-cycle inventory case study that quantifies the potential environmental benefits and burdens associated with “self-cleaning” surfaces, finally concluding that conventional approaches can be superior with respect to environmental impacts, when compared to self-cleaning surfaces [24]. However, it must be considered, that they compare the life-cycle of a self-cleaning surface based on a chemical coating cleaned only once to conventionally cleaned surfaces. Even though, this might be a realistic assumption for the assessed case study, it has to be noted, that a surface with a one-time “self-cleaning” functionality has to be seen as a rather poor example. This underpins the importance of a clear documentation of the basis of comparisons using life-cycle thinking tools. Therefore, great attention has been put on the documentation of the basis for the comparison in the study at hand.

**Figure 1:**
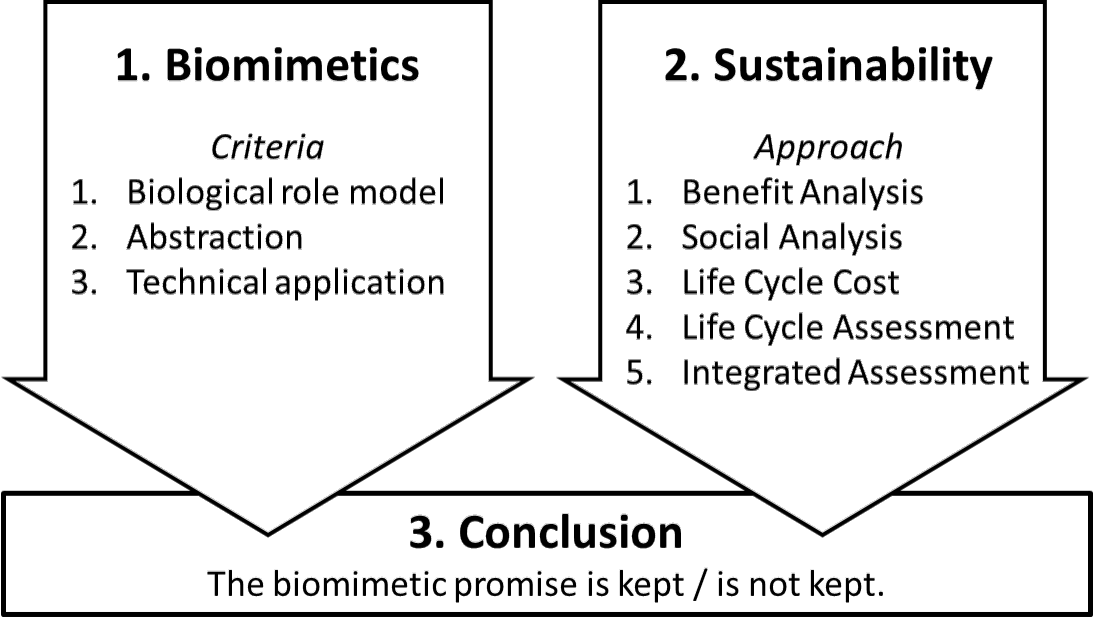
Three-stage validation procedure as to whether the biomimetic promise of an innovative biomimetic product is kept or not.

A possible approach to assess the implementation of the biomimetic promise has been suggested by Antony et al., based on a case study comparing a lightweight biomimetic ceiling structure from the late 1960s with two conventional up-to-date alternatives [16]. In a three-step process it is necessary to check first whether the product concerned is in fact a biomimetic innovation, and afterwards to analyse the sustainability of the product in terms of social, economic and environmental performance. Finally, it has to be decided whether the biomimetic promise is kept or not (Figure 1) [16].

Regarding the check whether the product is biomimetic, the approach refers to the criteria defined in the VDI Guideline 6220 Biomimetics – Conception and Strategy [13]. Regarding the assessment process as to whether a product contributes to sustainability or not, the product sustainability assessment (PROSA) approach, proposed by Grießhammer et al., has been applied [25] (see also http://www.prosa.org).

In this context, it is important to note, that sustainability can be measured in various ways according to the underlying concept of sustainability. However, some international standards are provided by ISO also being part of the PROSA. To guarantee comparability of the analyses and the results generated it is essential to use the same methodology.

In recent years, a number of research programs, starting with BIONA by the German Federal Ministry of Education and Research (BMBF) and the scholarship program “Bionics” by the German Federal Environmental Foundation (DBU) have been set up to promote further projects eventually resulting in biomimetic, more sustainable and innovative improved products.

Especially in the field of biomimetics in architecture sustainability aspects have been discussed [26]. The currently running Collaborative Research Center TRR 141 “Biological Design and Integrative Structures – Analysis, Simulation and Implementation in Architecture”, funded by the German Research Foundation (DFG), focuses on design and construction principles in biology and on their transfer to architecture and building construction. One of the topics addresses the investigation and validation of “the biomimetic promise: natural solutions as concept generators for sustainable technology development in the construction sector” (see http://www.trr141.de/).

Besides the expected results from the above Collaborative Research Center, and in order to reach defensible and sensible conclusions on the potential of biomimetic products to contribute to a more sustainable future, there is a need for further specific case studies based on sustainability assessments. A first approach to compare a biomimetic product with conventional alternatives using the PROSA approach has been performed by Antony et al. [16].

As a contribution to the discussion on sustainability in biomimetics, the present paper contains the second systematic product sustainability assessment of a biomimetic product. The product under investigation is one of the most widely known biomimetic products already available on the market, Lotusan^®^, a façade paint with self-cleaning properties.

## Results and Discussion

### Test of the criterion: Biomimetic product yes or no

As suggested by Antony et al. [16], clarifying whether the superhydrophobic properties of double-structured rough plant surfaces like the one of the sacred lotus (*Nelumbo nucifera*) have been abstracted from the biological model, and successfully applied to the façade paint Lotusan^®^ is the first step. This clarification serves as basis for deciding whether Lotusan^®^ has been defined correctly as biomimetic exterior paint. In the VDI guideline, the façade paint Lotusan^®^ has been explicitly examined by an interdisciplinary expert team and has been evaluated as fulfilling the preconditions in order to be called biomimetic [27].

#### Existence of a biological example

The first criterion requires that a biological model or precedent was found and studied by researchers and developers [13]. Based on results in basic biological research, the initial description of self-cleaning properties was done by the German biologist Wilhelm Barthlott in the late 1970s. This became the starting point for the development of Lotusan^®^ [28–29], a perfect example of a bottom-up process in biomimetics [1].

#### Understanding of the functional principle and abstraction

Within an abstraction phase the underlying functional principles of the biological model have to be translated into technology-compatible language [13,27]. In the present case the functional principle of the self-cleaning property of the lotus leaf can be considered as a precisely matched combination of a micro-rough plant surface, including hydrophobic waxes as part of the structuring and therefore is particularly suited to interact with water and causing the water to repel.

#### Existence of a technical realization

The existence of a technical realization, at least as a prototype, is the third and final criterion [13]. The result, the technical application of self-cleaning properties is what became well known as the lotus effect. The patent application was submitted on 25th July 1995 and granted by the German Patent and Trademark Office on February 4th in 1999 [8]. Sto SE & Co. KGaA (79780 Stühlingen, Germany) acquired the patent rights in 2009 and holds the trademark „Lotus-Effect^®^“. The fact that the façade paint Lotusan^®^ is available on the market since the late 1990s might be seen as a sufficient argument for the existence of a technical realization. The production of Lotusan^®^ is realized in a large-scale industrial process not significantly different from other paint production processes. This offers the desirable and at the same time rare opportunity to compare a biomimetic product with a conventional product on the same level of technology.

In summary, Lotusan^®^ fulfils all three criteria of a biomimetic product. However, the qualification by von Gleich with respect to the biomimetic promise needs a thorough case study. Therefore, we compared Lotusan^®^ with a conventional façade paint. Since self-cleaning properties can be realized in quite different ways, for a variety of applications and by using different materials, evaluations regarding sustainability have to be repeated for each individual case.

#### Product sustainability assessment (PROSA)

PROSA is an extensive sustainability assessment tool, which spans complete product life cycles and value chains. It assesses and evaluates the environmental, economic and social opportunities and risks of future development trajectories [25]. In the course of the present study, a comprehensive set of analysing techniques has been applied by using benefit analysis, social analysis, life-cycle cost assessment, life-cycle assessment, and life-cycle scenario analysis. According to the PROSA methodology, the results of the individual analyses are finally brought together within the framework of an integrated sustainability assessment [25].

With regard to the basic meaning of assessing the ecological performance as part of a product sustainability assessment, within the present study special emphasis has been put on assessing the ecological performance of the biomimetic façade paint Lotusan® within the course of a life-cycle assessment (LCA). But also economic effects regarding the life-cycle cost were considered to be of importance, and therefore have been analysed in depth. For PROSA, the consideration of possible social effects associated with the assessed products throughout the product life cycle is of great importance. Accordingly, the social effects regarding raw material provision have also been assessed within the framework of an orientative analysis, which is in line with the PROSA methodology.

The assessment of the sustainability of products or processes in absolute terms is not possible and not desirable either. Therefore, the sustainability of Lotusan^®^ has been assessed in comparison with the conventional façade paint Jumbosil^®^.

#### Basis for the comparison

In order to carry out the sustainability assessment of the façade paint Lotusan^®^ as part of a comparison, it is of great importance to accurately determine with reliable quality, which other façade paints might be considered as potential alternatives. In a first step, this means to identify the essential functions of façade paints. These are mainly to cover the building as an exterior layer, including all the various functions such as, for example, protection of underlying building layers against environmental effects like weather or radiation. In this connection, the water vapour permeability of the paint is a very important physical property.

The façade paints available on the market can essentially be divided into two main categories: First, those that focus on maximum gas exchange, which might positively influence the climatization of the building. However, in this case moisture may penetrate quicker and deeper into the construction, eventually resulting in serious damage to the building structure, especially in regions with higher amounts of precipitation. Secondly, those paints focusing on hydrophobicity or water-repellency to build up a secure barrier against the penetration of moisture into deeper layers of the building. In this case sufficient gas permeability needs to be secured. Lotusan^®^ was compared to a paint showing the same functional principle, providing a solid basis for the comparison of products.

Because a large number of façade paints is available on the market, Kougoulis et al. refer to about 20,000 products [30], the decision whether Lotusan^®^ should be compared to a generic paint formulation, typical for a hydrophobic façade paint available on the market, or to a real product, as a reference for typical façade paints available on the market had to be made. Suggestions made by Kougoulis et al. [30], may serve as a basis for a generic formulation, but the scoping phase revealed that considerations on an unknown formulation allow only rough approximations resulting in a serious increase of uncertainties having a negative influence on the informative validity of the study. Therefore, Lotusan^®^ was compared to Jumbosil^®^, a conventional paint from the same manufacturer. This turned out to be advantageous in many respects. Both paints are produced in the same production plant by the same staff using the same auxiliary and operating materials and the same production facilities. Furthermore, a good and consistent data basis was available for both paints, meeting the requirement of symmetry for comparison. Lotusan^®^ is available on the market for more than 10 years, facilitating the sustainability assessment of biomimetic products, which is challenged, for example, by the problem of defining a suitable reference product for comparison. Regarding the foresaid, a comparison between Lotusan^®^ and Jumbosil^®^ is largely unaffected by adverse effects. In addition, Jumbosil^®^ most probably is a suitable proxy for typical paints on the market.

A typical detached single-family house with a ceiling height of 2.4 m, has a façade surface of 250 m^2^ from which areas for windows and doors have to be deducted. Finally, for both options compared, a surface of 200 m^2^ requiring painting, were set as reference.

With regard to the use phase, the material safety data sheets for both paints were checked for differences during application of the paints and the utilization phase, for example, regarding special precautions because of toxicological reasons as well as information concerning the end-of-life treatment. In both cases no special precautions are required as both colours do not contain any hazardous or other substances to concern about with respect to use or exposition.

Based on information contained in the technical data sheets, and verified by the manufacturers both paints can simply be painted over. In both scenarios of our approach no damaged areas emerge throughout the façade paintings service life time, which would require further actions of conservation.

Since empty buckets and lids result from each painting, waste treatment of packaging materials had been taken into account. They have been recorded together with the efforts of the end-of-life treatment of the paint on the building at the end of the life cycle of the building. The end-of-life treatment has been assumed as a treatment with currently conventional disposal techniques, including the demolition of the building, the sorting of different material fractions, transport and final disposal in an inert landfill.

**Lotusan® (option 1):** Lotusan® is a water-based silicone resin façade paint. In accordance with the reporting guideline on the ingredients of decorative paints published by the association of the German paint and printing ink manufacturers (VdL), Lotusan^®^ consists of an emulsion of polyoxysiloxane, polymer dispersion, titanium dioxide, silicon dioxide, water and additives [31]. According to the technical bulletin [32], Lotusan^®^ is characterized by a density of 1.4–1.6 g/mL, and is highly permeable to carbon dioxide and water vapour. Furthermore, it also provides a high degree of natural protection against algae and fungal attack. Lotusan^®^ is extremely hydrophobic and shows no swelling. Contaminations like airborne dirt particles are washed away with the next rainfall.

**Jumbosil^®^ (option 2):** Jumbosil^®^ is a filled, silicone-annealed, dispersion-based façade paint. In accordance with the reporting guideline [31], Jumbosil^®^ consists of polymer dispersion, titanium dioxide, calcium carbonate, silicate fillers, talcum, water, glycol ether, aliphatic compounds, additives and preserving agents [33]. Jumbosil^®^ is characterized by a density of 1.5–1.6 g/mL and is, according to the technical data sheet, suitable for slightly filling, opaque exterior paintings on organic and mineral substrates. Jumbosil^®^ is water-repellent and permeable to carbon dioxide and water vapour.

**Comparing the material input for the formulation of Lotusan^®^ and Jumbosil^®^:** Primary data on the formulation of Lotusan® and Jumbosil® have been provided by the R&D-Section of Sto SE & Co. KGaA. A comparison of the formulation components is given in Table S1 (Supporting Information File 1).

Information on the physical composition of both façade paints, given as mass fractions, has been used for the modelling of the façade paint production. Where necessary, simplifications were employed and components reassigned. A first modelling setup has been checked in cooperation with chemical experts from Sto SE & Co. KGaA. In parallel, the case study by Kougoulis et al. [30] has been analysed to provide further evidence that the assumptions on the modelling of the formulation of the two paints are justified. Related to their mass, the most relevant formulation components of both paints are water, pigment, fillers and polymer dispersion.

The mass of TiO_2_ is about 2 times higher in Lotusan^®^ than in Jumbosil^®^. Similarly this applies to the 1.5 times higher water content of Lotusan^®^ compared to Jumbosil^®^´s water content. On the other hand, the content of polymer dispersion in Jumbosil^®^ is twice as high as the one in Lotusan^®^. The mass portion of fillers is 44% for Jumbosil^®^ and 34% for Lotusan^®^, while the mass portion of additives is comparable. Additionally, 4% of silicone resin and 1% of silicone-based hydrophobizing agent is part of the formulation of Lotusan^®^.

#### Benefit analysis

In the framework of benefit analysis, Lotusan^®^ und Jumbosil^®^ are compared to each other with regard to the benefit derived from their use as façade paintings. Façade paint has to meet a broad variety of aspects of utility. Aspects of utility can be of various kinds, such as functional, emotional or even societal in nature. A sole analysis of the functional utility would involve the danger of being blind for other aspects of utility that play a decisive role regarding the choice and use of products or product services. From the point of view of a consumer, a product always has a variety of additional, not functional-related aspects of utility, which arise from symbolic and maybe societal considerations. In this respect, the consumer choice on a the utility of a product might be seen as a multi-dimensional decision matrix, and it cannot be assumed that consumers only decide on the basis of sole considerations about the functional utility. Therefore, in the framework of a product sustainability assessment, aspects of utility are being analysed more intensively than this would be possible by carrying out an LCA study alone, where the benefit of a product is recorded slightly above the functional utility [16,25].

The functional utility of products can be usually clearly defined. Because essential elements of practical utility are measurable, these aspects can be well compared in comparative product tests [25]. The diligent determination of the functional utility as well as the thorough documentation of the determination process is applied in the course of classical LCA studies. The functional utility, in this context called functional unit, serves as a quantifiable reference quantity. With regard to the comparison of Lotusan^®^ and Jumbosil^®^, the functional utility can be defined as the provision of the outer layer of the building envelope and therefore also the protection of the building envelope against environmental influences. Both paints keep the deeper layers of the building envelope free from moisture, and can therefore contribute to maintaining the structural integrity of the building envelope. At the same time, both paints also have to allow for a sufficient level of permeability to gases such as carbon dioxide and oxygen.

Against the background of the above given description of functional utility, it can be stated that both paints are highly comparable. This also applies to the overall lifetime of the paints. According to information provided by the German Federal Ministry for the Environment, Nature Conservation, Building and Nuclear Safety, the expected product service life time of a silicone-based exterior painting is 15 years [34].

Regarding the super-hydrophobic properties of Lotusan^®^, together with its even higher permeability to carbon dioxide and oxygen compared to Jumbosil^®^, it can be assumed that Lotusan^®^ might reach a longer service life. First insights from assessments provided by the manufacturer suggest this [35–37]. According to [34], a service life of 20 years can be assumed for dispersion-silicate façade paintings. Therefore, in the study at hand, different periods of service life have been assessed: 20 years for Lotusan^®^ and 15 years for Jumbosil^®^. Because this might be a starting point for critics, it has been decided, in line with common practice of comparative product sustainability assessments, to also consider a shorter service life of only 15 years for a Lotusan^®^-based façade painting. Overall, it can be stated that both paints fulfil the practical utility in a similar way, albeit for different periods of time.

As mentioned before, the functional utility does not cover all utility dimensions that might be taken into account when choosing between different products. This applies even more in cases where there is a large degree of congruence in the practical utility. In such cases the symbolic utility and the societal utility gain importance for the decision-making process [16]. In addition to the functional properties, a façade paint obviously contributes significantly to the outward appearance of a building. Therefore, the façade painting can be seen as a representative and fundamental characteristic of a building. The façade’s purity, or even more its technical cleanliness, is of great importance for the façade’s perception of high optical quality. The optical quality of the paint is best at the time of façade painting. Throughout the paintings service life, impurities, for instance through rain and wind or even fouling caused by bacteria or fungal infestation lead to a progressive loss of optical quality of conventional façade paintings. In this respect, a major difference exists between the two compared façade paints. Because of Lotusan^®^’s pronounced hydrophobic characteristic, impurities and contaminations are washed away with the next rainfall. While the optical quality of conventional façade paintings decreases over the service life time, Lotusan^®^ has the potential to constantly maintain the original optical properties for a longer time-span or even over the entire product life cycle, providing an added value in terms of a constantly high level of optical quality compared to the optical quality of conventional façade paints decreasing over time.

It should be stressed in conclusion that both façade paintings satisfy the requirements of practical utility equally well, but maybe for different periods of time. With regard to the symbolic utility, an additional benefit has to be attributed to the Lotusan^®^ paint, since the self-cleaning effect enables it to constantly maintain the original optical quality over the entire service life span. The self-cleaning effect of a façade painted with Lotusan^®^ can therefore be seen as an added value immanent to the product and might be regarded as an added symbolic utility.

In order to be able to finally assess which product, over the entire observation period and with regard to all relevant aspects of utility, performs as the better product, a systematic and integrated evaluation is required. A robust quantification of the functional utility of the two compared products is at the same time the starting point and an indispensable basis for the implementation of both LCA and life-cycle cost assessment (LCC) as the relevant life-cycle thinking tools used throughout the study.

In addition to this and on the basis of the results of LCA and LCC, the effectiveness of possible measures likely to be able to make up the loss in optical quality of a Jumbosil^®^-based façade painting has been analysed. The implementation of such measures could result in a more or less functional equivalence of symbolic utility of the both compared façade paints. An in-depth analysis has been performed for two possible measures throughout this study. The potential influence of customer choices has been analysed as part of a comparative scenario analysis. On the one hand, an additional painting after 12.5 years has been considered. From a technical point of view this would be an early replacement or, in other terms, an arbitrary shortening of the service life time. On the other hand the efforts of a façade cleaning after 7.5 years have been analysed. This allows a quantification of the aspects of utility, depending on customer behaviour, even though this means quantifying “value choices“.

It should be noted that the results of the benefit analysis alone are insufficient to allow an accurate prioritization in favour of one of the façade paintings, at least until an added environmental or economic value of one of the two compared façade paints has been clearly shown during the course of the scenario analysis.

#### Social analysis

With regard to the production phase, the use phase and the final disposal at the end of the life cycle, no significant differences between the two façade paints could be identified. Regarding the provision of raw materials required for the two paints differences cannot be ruled out a priori. As there might be differences between the two paints, the bills of materials of the diverse formulation inputs have been checked.

The paint producer communicated on request that only such raw materials were used in the formulation of the paints investigated in the scope of this study that come from Germany or Europe. Concerning the provision of raw materials from Germany or Europe, it may be assumed that the relevant requirements for safety and health of workers and staff are met in this respect. While, against this background, the possibility that there is still potential for further improvement of safety at work cannot be strictly ruled out, it may be assumed that major differences relating to social effects between the two colours can be ruled out. Furthermore, it has been decided to also evaluate the ecotoxicity potential and the human toxicity potential related to the two product systems as part of the LCA. Using the respective indicators of the USEtox impact assessment model (see also Table S2, Supporting Information File 1) results are discussed within the framework of the discussion of LCA results.

In summary, significant social or societal differences or such relating to the health of customers were identified between the two façade paints, neither for production, nor for the processing at the site of the building or the disposal phase. The paints use very similar materials in their formulation. The above mentioned insights led to the conclusion that there no detailed analysis of social effects was needed within the framework of this case study.

#### Life-cycle cost assessment

A key part of a systematic and comprehensive product sustainability assessment is addressing the economic dimension of sustainability, as it can be assumed that the product-related cost is of great importance when customers have to decide between different products. The comparison of product-related cost takes into account the customer-related life-cycle cost. In this context, life-cycle cost is the cost that occurs throughout entire life cycle of the paints, also including the labour cost of painting the façade. Also of importance are operating expenditures, as for example expenditures for the heating of the building, and the cost arising from final demolition and disposal of the paints. Consequently, the comparison of the expected life-cycle cost covers the economical dimension as part of a product sustainability assessment.

In the scope of this study, both operating expenses and cost arising from final demolition and disposal are likely not to differ from each other with respect to the two paints. Both façade paints can be applied using the same technical equipment, and, as can be stated on the basis of information given by the product safety datasheet, the same occupational safety requirements can be applied to both of them [38–39]. The same applies to the end-of-life treatment.

The calculation of life-cycle cost is based on retail prices that are freely available in the internet. First of all, the average price per m^2^ in the field of professional façade painting was being sought. For reasons of transparency, many painting companies disclose their retail price calculation on the company website. On the basis of these publications and of further similar calculations, a retail price of about 20.00 € per m^2^ (including material cost) could be retrieved as suitable approximation regarding the German market and including delivery and installation of the scaffold, possible pre-treatments such as façade cleaning, the required amount of paint and also the dismantling and return of the scaffold. All labour cost, overhead cost and the profit margin are also included therein. Table 1 shows the calculation of life-cycle cost for both paints.

**Table 1:** Results for the calculation of cost based on retail prices.

type of cost^a^	unit	Lotusan^®^	Jumbosil^®^

materials cost/l	(€)	11.91	5.95
materials cost/m²	(€)	4.29	2.38
material cost/façade	(€)	858.00	476.00
material cost/functional unit	(€)	3,432.00	2,380.00
total cost/m² (work + materials)	(€)	21.91	20.00
total cost/façade (work + material)	(€)	4,382.00	4,000.00
total cost/functional unit^b^	(€)	17,528.00	20,000.00
work (total)	(€)	14,096.00	17,620.00
	(%)	80.4	88.1
materials (total)	(€)	3,432.00	2,380.00
	(%)	19.6	11.9

^a^Materials cost is the cost for the paints, while total cost represents the sum of materials cost and labour cost of a professional painter. ^b^Because of three required repaint coatings with Lotusan**^®^** instead of four repaint coatings required when using Jumbosil**^®^** the total cost per functional unit for Lotusan**^®^** is lower than for Jumbosil**^®^**.

As both paints can be applied using the same techniques, it can be assumed that there are no differences regarding labour cost, overhead and the profit margin of the painter. Consequently, the retail price of 20.00 € per m^2^ is made up of a fixed cost element for the execution of work, and the cost element of the façade paint itself. As Jumbosil^®^ stands as reference for a typical product on the market, a price of 20.00 € per m^2^ has been chosen as the retail price of Jumbosil^®^. For Jumbosil^®^, a retail price of 89.33 € per 15 litre bucket could be determined [40]. Assuming a consumption rate of 0.2 litres per m^2^, and a two-layer coating given in the product data sheet [33] the materials cost was calculated. Compared to Jumbosil^®^, Lotusan^®^ is the more costly product. For Lotusan^®^, the materials cost was calculated considering a retail price of 148.90 € per 12.5 litre bucket [40], assuming a consumption rate of 0.18 litres per m^2^ and a two-layer coating given in the product data sheet [32]. With regard to the entire façade surface of 200 m^2^, the Lotusan^®^-based façade painting is ca. 9% more expensive. Regarding the entire life cycle of 75 years, four repaint coatings have to be assumed for a Jumbosil^®^-based façade painting, while a Lotusan^®^-based façade painting only requires three repaint coatings. Consequently, the higher materials cost for a Lotusan^®^-based façade painting is more than compensated by the longer service life time, resulting in reduced overall materials consumption and lower labour cost. Cost savings over the entire building life cycle of 75 years sum up to 2,472 €.

It is important to note here, that some aspects have been neglected in the cost calculation given above. No discount, for example, has been taken into account for cost arising from repaint coatings to be carried out in the future. From the perspective of Lotusan^®^, this might be seen as a conservative assumption, as the Lotusan^®^-based façade painting has to be repainted one time fewer. Moreover, possible changes in materials and labour cost have also been neglected.

#### Life-cycle analysis

**Figure 2:**
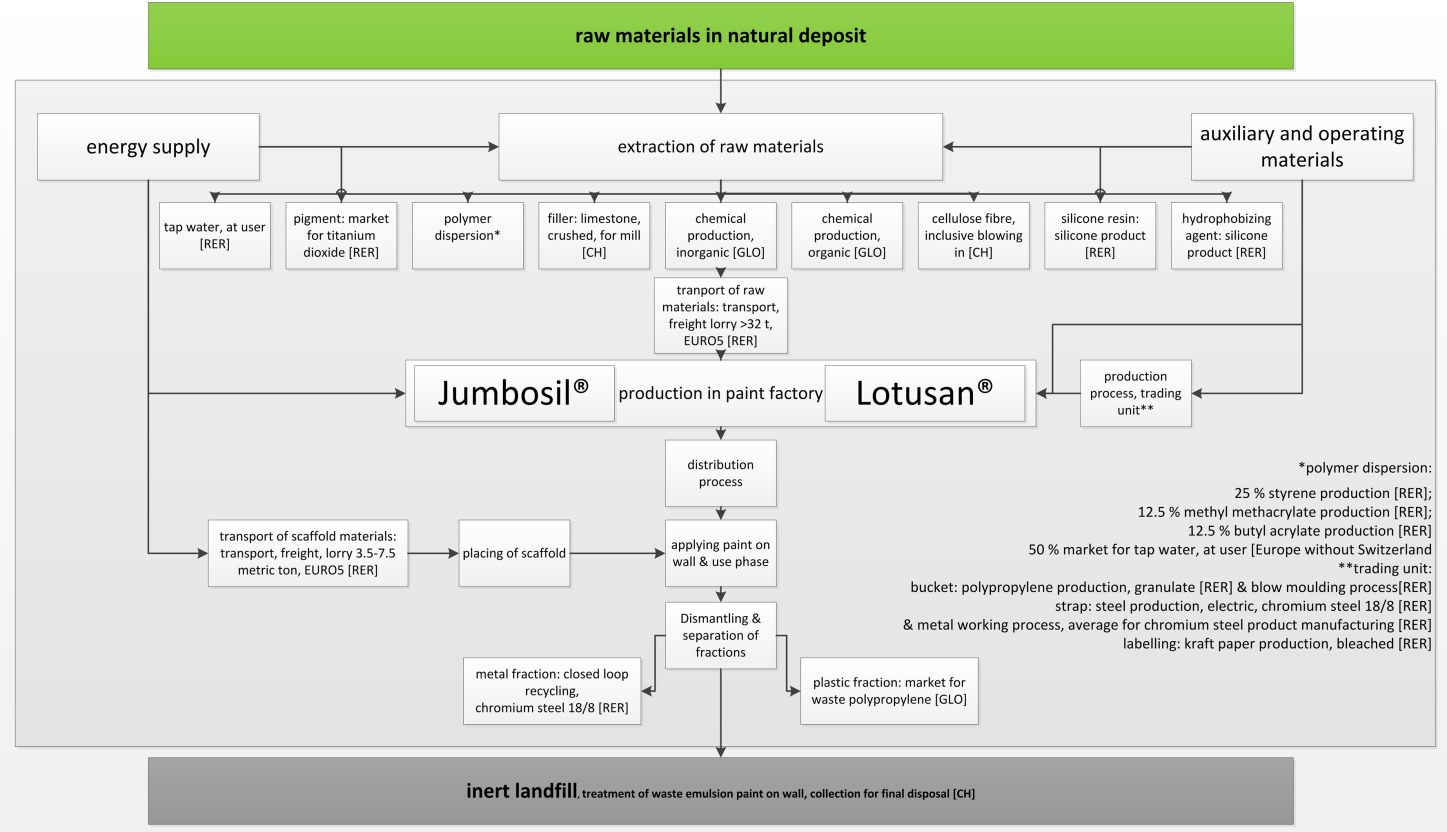
Schematic drawing of the system boundary with production and life-cycle steps for the two compared façade paints Lotusan^®^ and Jumbosil^®^ from cradle to grave.

For the purpose of this study, a comparative LCA has been carried out based on the standards DIN EN ISO 14040: 2006 [41] and DIN EN ISO 14044: 2006 [42]. In concrete terms, this means a comparison between the possible environmental impacts associated with painting a façade with Lotusan^®^ and Jumbosil^®^. The LCA was prepared “from cradle to grave” (Figure 2).

**Goal- and scope definition and modelling of the two alternative façade paintings:** The goal- and scope definition phase includes the determination of a functional unit (FU). In the present case the functional unit is equivalent to the functional utility, the provision of a structurally sound building through the provision of the outer layer of the building envelope. From this point of view, the two exterior paints can be considered as being equivalent in terms of equal functional utility and are directly comparable.

Throughout the 75 years life of the building, both a Lotusan^®^-based façade painting (three repainting coatings) and a Jumbosil^®^-based façade painting (four repaint coatings) have to be repainted. For this purpose, the production of the paints, their distribution to the building and the effort for assembling and dismantling the paint scaffold have to be taken into account. The software umberto (umberto NXT Universal © ifu hamburg 2013) has been used to prepare the LCA.

**Life-cycle impact assessment:** In the course of the life-cycle impact assessment (LCIA), potential environmental impacts of the two compared façade paints are determined by linking the LCI results, namely materials flow and energy flow, to specific environmental impact categories.

According to the requirements of [41], the selection of impact categories has to be done according to the goals of the study. In the framework of this study, a broad set of impact categories has been evaluated. The aim was to ensure that all relevant environmental issues are covered by the selection of impact categories. Likewise, a special emphasis has been put on selecting such impact categories providing maximum transparency and representing the scientific state of the art.

Result monitoring was carried out by using a combination of four environmental impact assessment models, including the cumulative energy demand (CED_fossil_, CED_nuclear_, CED_non-renewable_ as sum of the aforementioned), the ReCiPe environmental impact assessment model in the version of 2008, the IPCC method regarding the global warming potential (GWP) and the USEtox model for dealing with toxicology aspects related to the production and use of the two paints. A short description of the investigated categories is given in Table S2 (Supporting Information File 1). For an elaborate discussion of the different impact categories, we also refer to [43–44].

**Results of the Life Cycle Impact Assessment:** Table 2 shows the overall results for the two compared products both as absolute values as well as in terms of their share with respect to Lotusan^®^. All values are related to the functional unit of the provision of a façade painting of 200 m^2^ over 75 years.

**Table 2:** Overall results of the life-cycle impact assessment shown for each impact category.

impact indicator	abbreviation^a^	unit	Lotusan^®^	Jumbosil^®^	Jumbosil^®^/Lotusan^®^

cumulative non-renewable energy demand	CED_non-renewable_	[GJ]	9.3	14.3	1.54
global warming potential	GWP_100a_	[kg CO_2_-e]	645	893	1.39
water depletion potential	WDP	[m^3^]	4.82	3.78	0.78
terrestrial acidification	TAP_100a_	[kg SO_2_-e]	2.90	4.07	1.40
freshwater eutrophication	FEP	[kg P-e]	0.17	0.18	1.05
marine eutrophication	MEP	[kg N-e]	0.17	0.21	1.24
photochemical ozone formation potential	POFP	[kg NMVOC]	2.48	3.20	1.29
agricultural land occupation potential	ALOP	[m^2^a]	32.42	30.54	0.94
human toxicity, total	USEtox_humantox_	CTU	7.52·10^−5^	7.65·10^−5^	1.02
ecotoxicity, total	USEtox_ecotox_	CTU	1448	1205	0.83
particulate matter formation potential	PMFP	[PM10-e]	1.28	1.43	1.12

^a^A description of the impact indicators used in this study is found in Table S2 (Supporting Information File 1).

Results of the life-cycle impact assessment show that both paintings differ with regard to their potential environmental impact. Regarding the cumulative energy demand from non-renewable energy sources, the biomimetic product Lotusan^®^ has a lower indicator value. Jumbosil^®^ as the reference product has a 1.54-fold higher demand on non-renewable primary energy, which is mainly due to the higher share of polymer dispersion within the product formulation. A comparable situation is given with regard to the global warming potential (GWP). Jumbosil^®^ has a GWP that is about a factor of 1.39-fold higher than that of Lotusan^®^. The higher share of polymer dispersion within the product formulation of Jumbosil^®^ is the main reason also for the higher product related terrestrial acidification potential (TAP), the marine eutrophication potential (MEP) and the particulate matter formation potential (PMFP). However, freshwater depletion potential (WDP) and USEtox_ecotox_ are considered, Jumbosil^®^ achieves the lower values. This is mainly because of Lotusan^®^’s higher content of titanium dioxide (TiO_2_). Regarding the remaining impact indicators both façade paints are equal within the calculation inaccuracy.

**Contributions by life-cycle stages:** The key issue of the contribution analysis is a clarification of the composition of the overall results and the identification of the processes that have the greatest influence on the overall results. A first analysis investigates the contributions by life-cycle stages along the entire service life cycle of the two products (Table S3, Supporting Information File 1). For both façade paints, the contributions by the provision of the required raw materials are most important. This life-cycle stage contributes from 56% regarding CED up to 91% regarding USEtox_ecotox_ to the overall indicator results of Lotusan^®^. Regarding Jumbosil^®^, contributions reach from 59% (GWP, NMVOC) up to 85% of the overall USEtox_ecotox_ indicator result. An exception was found for Jumbosil^®^ with regard to the ALOP. Here, the provision of raw materials contributes only about 40% to the overall indicator result. With regard to the ALOP, the production and provision of packaging materials are more important (Lotusan^®^ 27%, Jumbosil^®^ 41%) than for other impact categories.

Besides the provision of raw materials, the contributions by the use phase are also worth mentioning. With the exception of WDP (Lotusan^®^ 4%; Jumbosil^®^ 6%), the contribution to all impact categories are at a level of at least around 10% or even higher. Regarding CED and GWP, the use phase contributes from 21% (CED, Jumbosil^®^) to 26% (GWP, Lotusan^®^) to the overall indicator results.

Due to the large contribution from the provision of raw materials, an in-depth analysis on the shares of the different raw materials has been carried out (Table S4, Supporting Information File 1). For both paints, the main contributions arise from the provision of TiO_2_ as white pigment. As already shown in Supporting Information Table S1, Lotusan^®^ has a higher TiO_2_ content (20%, compared to only 10% for Jumbosil^®^). Remarkable in this context are the results for the two USEtox indicators. They show that over 97% of the ecotoxicity potential related to the provision of raw materials required for Lotusan^®^ trace back to the provision of TiO_2_. The overall indicator result for USEtox_ecotox_ is dominated by the TiO_2_ provision, contributing more than 88% of the overall result for Lotusan^®^. The manufacturing phase and, in particular, the use phase are of only limited importance for this result.

Besides TiO_2_, the provision of the polymer dispersion is likewise of some importance. This applies even more to Jumbosil^®^, due to the higher content of polymer dispersion. Regarding CED und GWP, the provision of polymer dispersion contributes about 55% to the overall indicator result of Jumbosil^®^. With regard to Lotusan^®^, the contribution by the silicone resin is also not negligible, making up 7–10% of the indicator results. Regarding ALOP, silicone resin contributes even about 20%.

#### Scenario analysis

The three scenario analyses described in the following have been calculated on the basis of considerations resulting from the benefit analysis. Within the base-case scenario, a service life of 20 years has been assumed for a Lotusan^®^-based façade painting, taking into account the additional properties of Lotusan^®^ and its capacity to maintain the initial optical quality over the entire service life cycle. The assumption on the possible service life time is relevant for the results. Hence, both a reduced and an expanded service life time for a Lotusan^®^-based façade painting have been assessed.

With regard to the Jumbosil^®^-based façade paintings, two measures are described below that might at least temporarily be suitable in order to reach or regain equivalent optical quality. The scenarios do not aim at suggesting what customers should decide for, but it is the aim to show the implicit effects of a possible choice of customer. Results for all scenario analyses are given in Table 3.

**Table 3:** Overall results of the scenario analyses^a,b^.

impact category	Lotusan^®^	Jumbosil^®^	S1: Lotusan^®^	S2: Lotusan^®^	S3: Jumbosil^®^	S4: Jumbosil^®^
	service life: 20 years	service life: 15 years	service life: 15 years	service life: 25 years	service life: 12.5 years	service life: 15 years plus additional façade cleaning after 7.5 years

CED_non-renewable_	100%	154%	125%	75%	184%	190%
GWP_100a_	100%	139%	125%	75%	166%	172%
WDP	100%	78%	125%	75%	94%	192%
TAP_100a_	100%	140%	125%	75%	168%	162%
FEP	100%	105%	125%	75%	126%	128%
MEP	100%	124%	125%	75%	149%	144%
POFP	100%	129%	125%	75%	155%	164%
ALOP	100%	94%	125%	75%	113%	106%
USEtox_humantox_	100%	102%	125%	75%	122%	128%
USEtox_ecotox_	100%	83%	125%	75%	100%	92%
PMFP	100%	112%	125%	75%	135%	138%
Overall cost	100%	114%	125%	75%	137%	—^c^

^a^A description of the scenario analyses and of the parameters varied in S1 to S4 is given in the main text. ^b^Percentage values are normalized with respect to Lotusan**^®^** (set as 100%); higher values as 100% mean higher overall environmental impacts or higher cost. ^c^Because of lacking data on the typical cost for professional façade cleaning, no overall cost have been calculated for scenario S4.

**Service life:** (S1 Lotusan^®^) – Service life reduction using Lotusan^®^ painting for 15 years instead of 20 years: Within the base-case scenario, a service life of 20 years has been assumed for Lotusan^®^-based façade paintings. As this assumption is result-relevant and may feed criticism, a service life time of 15 years has been evaluated in analogy to the base-case assumption for Jumbosil^®^-based façade paintings. In this case, for the Lotusan^®^-based façade paintings as well four repainting coatings have to be taken into account. This results in a higher materials demand and in additional efforts in the course of the painting itself.

(S2 Lotusan^®^) – Service life expansion using Lotusan^®^ paint for 25 years instead of 20 years: Both in terms of structural and functional properties as well as for providing sufficient optical quality, a possible 5 year service life expansion of Lotusan^®^, thus reaching an overall service life time of 25 years, has been evaluated.

(S3 Jumbosil^®^) – The effect of repainting the Jumbosil^®^-based façade paint after 12.5 years due to a loss of optical quality: In this scenario, the effect of a repainting of Jumbosil^®^ after 12.5 years due to a loss of optic quality has been calculated. In technical terms, this can be seen as a customer-chosen reduction of service life time by 2.5 years (16%). Such customer behaviour is well known for example within the field of information and communications technology equipment, and has been recently described as “psychological obsolescence” [45]. A reduced product service life time of 12.5 years results in one additional painting over the 75 year life time of the building. Therefore, the environmental burden of one additional painting of the façade with Jumbosil^®^ has been evaluated.

(S4 Jumbosil^®^) – Consideration of a professional façade cleaning of the Jumbosil^®^-based façade paint after 7.5 years: A professional façade cleaning might be able to temporarily stop and reduce the continuing loss of optical quality. Although this is not the same as the ability of constantly maintaining the optical quality at a high level by self-drying façade paint, customers might consider it satisfactory. In contrast to scenario S3 given above, a cleaning of the façade could lead customers to take advantage of the complete service life of 15 years. In general, a façade cleaning can be performed either using a mobile platform, or alternatively by putting up a scaffold. In both cases it is assumed that a lorry transport is needed to put either the elevating platform or the scaffold to the building. In particular, a transport distance of 20 km has been taken into account. Regarding the façade cleaning process itself, the demand of energy and freshwater for running a high-pressure cleaner have been taken into account, assuming a power consumption of 1.5 kW, a water demand of 350 litres per hour, and an assumed time requirement of 3 h per 200 m^2^ façade. It is furthermore assumed that the façade cleaning can be done solely with clear water without detergents or other cleaning agents.

#### Integrated assessment and discussion

So far, all relevant dimensions of sustainability have been investigated within the course of the comparative product sustainability assessment independently of one another. However, dependencies exist between the individual PROSA tools applied in this comparison that need to be taken into consideration when discussing and interpreting the results [25]. This is especially true considering the challenge of bringing together the several findings of the individual analyses carried out within the product sustainability assessment (Table 4). It has been decided to do the integrated assessment at a qualitative level as this yields maximum transparency of the results. While single numerical score values are better comparable, they do not offer a corresponding transparency. For a discussion of advantages and disadvantages of doing the integrated assessment at a quantitative or a qualitative level, we refer to the discussion in [16].

**Table 4:** Overview of the results for the individual analyses carried out within the product sustainability assessment.

analysis tool		Lotusan^®^	Jumbosil^®^

check on biomimetic product		biomimetic	—
benefit analyses	functional utility	Both paints fulfil the functional utility in a similar way, albeit for different periods of time. For Lotusan^®^-based façade paintings a 20 year service life is assumed, due to higher product qualities in terms of wettability and gas exchange.	Both paints fulfil the functional utility in a similar way, albeit over varying periods of time. For Jumbosil^®^-based façade paintings a 15 year service life is assumed.
	symbolic utility	Preservation of optical quality over the life cycle is an additional aesthetic value.	—
social life-cycle assessment	orienting analysis	No fundamental differences are expected (consequently no in-depth analysis was carried out).	
life-cycle cost assessment	operating expenses	No fundamental differences are assumed.	
	cost for demolition and final disposal	No fundamental differences are assumed.	
	overall materials cost	A Lotusan^®^-based façade painting is more expensive by 1.91 €/m^2^. A service life of 20 years was taken into account.	
	labour cost	No fundamental differences are expected due to information given in the TDS; labour causes 81% of overall cost.	No fundamental differences are expected due to information given in the TDS; labour causes 88% of overall cost.
	overall cost	In absolute terms, the cost of a Lotusan^®^-based 200 m^2^ façade painting are 4,382 € and therefore by 382 € more expensive than a Jumbosil^®^-based façade painting of the same dimensions. Cost are more than compensated by the longer service life time, resulting reduced overall materials demand and lower labour cost (only 3 instead of 4 repaintings). Cost savings over the entire building life cycle of 75 years sum up to 2,472 €.	The provision of a Jumbosil^®^-based façade painting is about 91% of the cost compared to the provision of a Lotusan^®^-based façade painting. The overall cost is strongly related to the assumed product service life times (see also results of scenario analyses below).
life-cycle assessment	CED, GWP, TAP, MEP, POFP, PMFP	Considering a life time of 75 years of the building, a Lotusan^®^-based façade painting might be advantageous compared to a Jumbosil^®^-based façade painting.	Considering a life time of 75 years of the building, the values for a Jumbosil^®^-based façade painting are about 10–54% higher than for a Lotusan^®^-based façade painting.
	FEP, ALOP, USEtox_humantox_	Both façade paints lie within a similar range.	
	WDP, USEtox_humantox_	Values for a Lotusan^®^-based façade painting are about 20–28% higher than for a Jumbosil^®^-based façade painting.	A Jumbosil^®^-based façade painting might be advantageous compared to a Lotusan^®^-based façade painting
scenario analyses	S1: Service-life reduction of Lotusan^®^	25% increase of overall LCA and cost results. Lotusan^®^ is a little more advantageous compared to Jumbosil^®^ regarding CED, GWP and TAP. Regarding MEP and POFP both paints compare are at a comparable level. Regarding FEP, ALOP, USEtox and PMFP, indicator values for a Lotusan^®^-based façade painting are about 20–25% higher than for a Jumbosil^®^-based façade painting. Regarding WDP values are about 60% higher.	—
	S2: service-life expansion of Lotusan^®^	25 % reduction of overall LCA and cost results. Lotusan^®^ is more advantageous compared to Jumbosil^®^ in nearly all LCIA indicators and cost. Regarding WDP and USEtox_ecotox_ both paints perform at a comparable level.	—
	S3: service-life reduction of Jumbosil^®^	—	Regarding CED, GWP, TAP, MEP, POFP, PMFP and also overall cost the existing gap widens. Regarding FEP, ALOP and USEtox_humantox_ the result for Jumbosil^®^ is now negative. Regarding WDP and USEtox_ecotox_ the advantage of Jumbosil^®^ is lost.
	S4: Additional façade cleaning of the Jumbosil^®^-based façade painting	—	Regarding CED, GWP and TAP, MEP, POFP and PMFP the existing gap widens. Regarding ALOP and USEtox_ecotox_ the advantage of Jumbosil^®^ is lost. Regarding WDP, FEP and USEtox_humantox_ the result for Jumbosil^®^ is now negative.

As has been found within the course of the benefit analysis, both paints fulfil the requirements of functional utility equally well, albeit for different periods of time (Lotusan^®^ 20 years, Jumbosil^®^ 15 years). Furthermore, Lotusan^®^ has a particular added value with respect to the symbolic utility. The maintaining of the initial optical quality at a constantly high level over the entire product life cycle is something a Jumbosil^®^-based façade paint does not yield. From the perspective of the benefit analysis an additional benefit can be established for the Lotusan^®^-based façade painting.

The comparison has also been done regarding possible social effects. Thereby, both the bill of materials and the product safety data sheets of the final products have been checked for possible differences, with the result that no substantial differences exist with regard to the handling and disposal of the final products. Concerning the provision of raw materials it has been decided to additionally evaluate the products regarding toxicology aspects within the LCA. In the course of conducting the LCA, the demand for titanium dioxide in both paints could be identified as main driver of toxicity potential indicator results. Regarding the base-case scenario, Lotusan^®^ shows about 20% higher USEtox_humantox_ indicator results, arising from the higher content of titanium dioxide. With regard to the USEtox_ecotox_ indicator result, it can be established that the longer product service life of a Lotusan^®^-based façade painting counterbalances the additional demand for titanium dioxide. It should be noted that, taking into account the same service life time for both façade paintings (as analysed in scenario S1), indicator values for a Lotusan^®^-based façade painting are about 20–25% higher than for a Jumbosil^®^-based façade painting. Due to the fact that, according to the manufacturer, in both paints only raw materials from Europe were used in the product, it could have been ruled out that major differences exist in terms of social effects between the two paints compared within this study. As regards the safety of the final products, based on the information given in the product safety datasheets, it can be clearly stated that both paints do not contain any hazardous substances or substances the use of which and the exposition to which would cause concerns. From the point of view of the customers, they can therefore be seen as equally safe alternatives.

Regarding the life-cycle cost, Lotusan^®^ is the more expensive product, but at the same time the higher investment cost for a Lotusan^®^-based façade painting are more than compensated by the longer service life time, resulting in reduced overall materials demand and lower labour cost (only three instead of four repaint coatings). Cost savings over the entire building life cycle of 75 years sum up to 2,472 € (ca. 10%). The cost saving is clearly related to the estimated longer service life of a Lotusan^®^-based façade painting. Taking into account the service life reduction of Lotusan^®^ (scenario S1), the overall cost of a Lotusan^®^-based façade painting are correspondingly slightly higher (ca. 10%).

In terms of the life-cycle impact assessment, results differ for the two façade paints. While Lotusan^®^ performs better regarding the majority of impact indicators (CED, GWP TAP, MEP, POFP, and PMFP), Jumbosil^®^ performs better regarding WDP, USEtox_humantox_. Regarding FEP, ALOP and USEtox_ecotox_, both façade paints more or less lie within the same range.

In total, it can be ascertained that there are differences between the two paints compared. However, these differences are relatively minor in terms of the provision of raw materials and the production of the paints. The substantial differences between the paints arise from the service life of the façade paintings. This can be shown, for example, with regard to the findings of scenario S1 where the same number of repaint coatings for the entire life cycle of 75 years has been assessed for both Lotusan^®^ and Jumbosil^®^. Results of scenario S1 show that Lotusan® is still a little more advantageous compared to Jumbosil^®^ in terms of CED, GWP and TAP, while for FEP, ALOP, USEtox and PMFP the indicator values for a Lotusan^®^-based façade painting, are about 20–25% higher than for a Jumbosil^®^-based façade painting. For the majority of impact indicators it can be stated that the differences between the two façade paintings lie within the range of one additional repaint coating over the entire life time of the building of 75 years.

## Conclusion

Within the course of the systematic product sustainability assessment at hand, the biomimetic façade paint Lotusan^®^ has been compared to the conventional façade paint Jumbosil^®^. The Lotus-Effect^®^ technology has been successfully put into practice in Lotusan^®^ which is one of the best known and most widely used biomimetic products. Several aspects such as benefit analysis, LCC, LCA and scenario analysis have been addressed within the product sustainability assessment. Because of the outstanding functional utility of the product it can be seen as a useful measure of protecting a building against weathering. Furthered by the experimentally determined additional functionalities, a bigger service life time of 20 years has been ascribed to a Lotusan^®^-based façade painting. In conclusion, the biomimetic façade paint Lotusan^®^ has been identified as a cost-effective and resource-saving product. Lotusan^®^-based façade paintings have a comparatively low overall impact on the environment. Furthermore, it could be reliably determined that the use of the product has no negative effect in terms of toxicology and social aspects.

Results of both LCC and LCA are highly dependent on the possible behaviour of customers. The differences in functional utility might justify a different behaviour of customers. In a few years, when some of the first reference objects will reach the estimated end of the first service life cycle, it will be interesting to see whether the estimated service life extension of five years of Lotusan^®^-based façade paintings constitutes an objectively justified presumption. Should it prove to be true, Lotusan^®^ turns out as the favourable product, both in terms of economic performance and in terms of ecological relevance. It can therefore be asserted that with regard to Lotusan^®^ the biomimetic promise is kept.

## Supporting Information

File 1 Detailed data.
