# Supplementary material for: The cleaner, the greener? Product sustainability assessment of the biomimetic façade paint Lotusan® in comparison to the conventional façade paint Jumbosil®
Source: Beilstein J Nanotechnol. 2016 Dec 29;7:2100–15. doi: 10.3762/bjnano.7.200 (PMC5238657; doi:10.3762/bjnano.7.200)
Supplement: File 1 — Detailed data. [file Beilstein_J_Nanotechnol-07-2100-s001.pdf]

Supporting Information

for

The cleaner, the greener? Product sustainability assessment of the biomimetic façade paint Lotusan® in comparison to the conventional façade paint Jumbosil®

Florian Antony[1,2,3], Rainer Grießhammer[2], Thomas Speck[1,3,4,5] and Olga Speck*[1,3,5]

Address: [1]Plant Biomechanics Group, Botanic Garden, Faculty of Biology, University of Freiburg, 79104 Freiburg, Germany, [2]Öko-Institut e.V., Institute for Applied Ecology, 79017 Freiburg, Germany, [3]Competence Network Biomimetics, Germany, [4]Freiburg Materials Research Center (FMF), 79104 Freiburg, Germany and [5]Freiburg Centre for Interactive Materials and Bioinspired Technologies (FIT), 79110 Freiburg, Germany

Email: Olga Speck* - olga.speck@biologie.uni-freiburg.de

* Corresponding author

Detailed data

Table S1: Comparison of the bill of formulation components for the two façade paints Lotusan® and Jumbosil®.

**Material**	**Lotusan®** **[mass %]**	**Jumbosil®** **[mass %]**	**Used dataset (ecoinvent V3.01)** [1]
Water-based polymer dispersion; 50 % styrene-acrylic	10	23.8	25 % styrene production [RER] + 12.5 % methyl methacrylate production [RER] + 50 % market for tap water, at user [Europe without Switzerland] + 12.5 % butyl acrylate production [RER]
Pigment, titanium dioxide (TiO_2)	20	10	Market for titanium dioxide [RER]
Filler (rock flour)	34.4	44.4	Limestone production, crushed, for mill [CH]
Aliphatic solvents	0.5	0.9	50% chemical production, inorganic [GLO] + 50 % chemical production, organic [GLO]
Glycol ether	---	1	50% chemical production, inorganic [GLO] + 50 % chemical production, organic [GLO]
In-can preservation	---	0.6	50% chemical production, inorganic [GLO] + 50 % chemical production, organic [GLO]
Film preservation	---	0.4	50% chemical production, inorganic [GLO] + 50% chemical production, organic [GLO]
Dispersing agent	0.1	0.4	50% chemical production, inorganic [GLO] + 50% chemical production, organic [GLO]
Defoaming agent	0.2	0.2	50% chemical production, inorganic [GLO] + 50% chemical production, organic [GLO]
Methyl cellulose	0.1	0.2	50% chemical production, inorganic [GLO] + 50% chemical production, organic [GLO]
Thickener	0.5	0.2	50% chemical production, inorganic [GLO] + 50% chemical production, organic [GLO]
Water	28.2	38.3	Tap, water at user [RER]
Hydrophobizing agent	1	---	Silicone product production [RER]
Fibres (cellulose)	1	---	Cellulose fibre production, inclusive blowing in [CH]
Silicone resin	4	---	Silicone product production [RER]
Total	100	100	

Table S2: Environmental impact categories observed within this study.

**Valuation System**	**Impact Indicator**	**Abbre-viation**	**Unit**	**Short Description**	**Source/ citation**
**Cumulative Energy Demand**	Cumulative Fossil Energy Demand	CED_{fossil}	MJ	Summarizes the total energy input of all fossil forms of energy as a value or primary energy demand. The CED_{fossil} is given in MJ.	[2]
	Cumulative Nuclear Energy Demand	$CED_{nuclear}$	MJ	Summarizes the total energy input of nuclear energy as a value or primary energy demand. The $CED_{nuclear}$ is given in MJ.	
	Cumulative Non-renewable Energy Demand	$CED_{non\text{-}renewable}$	MJ	Summarizes the total energy input of all not renewable forms of energy as a value or primary energy demand. The $CED_{non\text{-}renewable}$ is given in MJ.	
**IPCC 2007**	100 years Global Warming Potential	GWP_{100a}	kg CO_2e[a]	Based on the anthropogenic greenhouse gas emissions. All greenhouse gas emissions are summed up, whereby the gases' specific Global Warming Potential is expressed in comparison to the CO_2. The GWP is given in units of kilograms of CO_2 equivalent, which means that amount of CO_2 that has the same degree of effectiveness as another greenhouse gas.	[3]
**ReCiPe Midpoint (H) w/0 LT**	Water Depletion Potential	WDP	m³	Freshwater is not only a scarce resource in many regions; it is an indispensable prerequisite for many organisms. Furthermore, freshwater can be seen as a renewable abiotic resource, that is irreversible exhausted only in few processes (i.e. hydrolysis of cement in concrete production). The impact indicator WDP regards the usage of water in terms of used water volume. WDP is an life cycle inventory indicator, with an only formal characterization factor of 1m³/m³ for all types of water, regardless of its source and for example local or regional scarcities.	[2]
	Terrestrial Acidification w/o LT, w/o LT	TAP 100	kg SO_2e[1]	A lowering of the soil pH value is mainly caused by sulphur dioxide, ammonia, and nitrogen oxides. Sulphur dioxide is the key indicator for the quantification of the acidification potential. All other emissions with a pH value lowering effect are therefore related to the effectiveness of sulphur dioxide equivalents.	
	Freshwater Eutrophication Potential	FEP	kg Pe[1]	Describes the potential for excessive accumulation of freshwaters and is also called "over-fertilization" potential. The FEP is given as kg phosphor equivalent	
	Marine Eutrophication Potential	MEP	kg Ne[1]	Describes the potential for excessive accumulation of marine waters and is also called "over-fertilization" potential. The MEP is given as kg nitrogen equivalent	
	Photochemical Ozone Formation Potential	POFP	kg NMVOC	As a result of chemical reactions of nitrogen oxides with volatile organic compounds under the influence of ultraviolet light, ozone results from photochemical reaction in near-ground air layers. The key indicator is defined as kg non-	

Valuation System	Impact Indicator	Abbre-viation	Unit	Short Description	Source/ citation
				methane volatile organic compound.	
	Agricultural Land Occupation Potential	ALOP	m²*a	The indicator Agricultural land occupation potential adds up to the amount of agriculturally used land, needed to fulfil the function of an investigated product system. It is expressed in the unit square meter multiplied with one year as a time factor.	
	Particulate Matter Formation Potential	PMFP	kg PM10e[1]	Describes the Potential of a product or product system to build Particulate Matter. It is expressed in the unit PM10 equivalents which mean particles with an average diameter of about 10 µm.	
**USEtox**	Human Toxicity, total	$USEtox_{humantox}$	CTU_h	The USEtox human toxicity potential is expressed in comparative toxic units (CTU_h), the estimated increase in morbidity in the total human population, per unit mass of a chemical emitted. The indicator CTU_h per kg emitted can also be described as disease cases per kg emitted.	[2]
	Ecotoxicity, total	$USEtox_{ecotox}$	CTU_e	The USEtox ecotoxicity potential (aquatic ecotoxicity impacts) is also expressed in comparative toxic units (CTU_e), an estimate of the potentially affected fraction of species (PAF) integrated over time and volume per unit mas of a chemical emitted.	

[a]The letter “e” stands for “equivalent”.

Table S3: Contribution to impact categories by life cycle stages for the two compared paints given as absolute values and in terms of the relative share of each overall result.[a]

Impact category [Unit]	Façade paint	Bill of Materials		Packaging		Production		Distribution		Use Phase		End of Life-Treatment		Sum	
		abs.	rel.	abs.	rel.	abs.	rel.	abs.	rel.	abs.	rel.	abs.	rel.	abs.	rel.
$CED_{non\text{-}renewable}$ [MJ]	Lotusan®	5.2E+03	55.8%	6.7E+02	7.2%	2.8E+02	3.0%	6.7E+02	7.1%	2.5E+03	26.3%	4.8E+01	0.5%	9.3E+03	100.0%
	Jumbosil®	8.9E+03	62.1%	9.3E+02	6.5%	4.0E+02	2.8%	9.5E+02	6.6%	3.1E+03	21.4%	6.9E+01	0.5%	1.4E+04	100.0%
GWP_{100a} [kg CO_2e]	Lotusan®	3.7E+02	57.0%	2.4E+01	3.7%	1.8E+01	2.7%	4.1E+01	6.4%	1.6E+02	24.4%	3.8E+01	5.9%	6.4E+02	100.0%
	Jumbosil®	5.2E+02	58.7%	3.4E+01	3.8%	2.5E+01	2.8%	5.9E+01	6.6%	2.0E+02	22.0%	5.4E+01	6.1%	8.9E+02	100.0%
WDP [m^3]	Lotusan®	4.3E+00	89.5%	7.5E-02	1.5%	1.7E-01	3.5%	4.6E-02	0.9%	2.0E-01	4.1%	1.7E-02	0.4%	4.8E+00	100.0%
	Jumbosil®	3.1E+00	81.9%	1.0E-01	2.7%	2.5E-01	6.5%	6.5E-02	1.7%	2.5E-01	6.5%	2.4E-02	0.6%	3.8E+00	100.0%
TAP_{100a} [kg SO_2e]	Lotusan®	2.2E+00	74.4%	8.8E-02	3.0%	2.4E-02	0.8%	1.4E-01	4.8%	4.8E-01	16.5%	1.4E-02	0.5%	2.9E+00	100.0%
	Jumbosil®	3.1E+00	76.1%	1.2E-01	3.0%	3.4E-02	0.8%	2.0E-01	4.9%	6.0E-01	14.7%	1.9E-02	0.5%	4.1E+00	100.0%
FEP [kg Pe]	Lotusan®	1.2E-01	72.2%	6.3E-03	3.8%	2.0E-02	11.7%	4.6E-03	2.7%	1.6E-02	9.5%	1.8E-04	0.1%	1.7E-01	100.0%
	Jumbosil®	1.1E-01	63.9%	8.7E-03	5.0%	2.8E-02	16.0%	6.5E-03	3.7%	2.0E-02	11.2%	2.5E-04	0.1%	1.8E-01	100.0%
MEP [kg Ne]	Lotusan®	1.3E-01	76.3%	4.2E-03	2.4%	5.8E-03	3.3%	7.0E-03	4.1%	2.3E-02	13.3%	9.7E-04	0.6%	1.7E-01	100.0%
	Jumbosil®	1.6E-01	74.7%	5.8E-03	2.7%	8.3E-03	3.9%	1.0E-02	4.7%	2.9E-02	13.4%	1.4E-03	0.7%	2.1E-01	100.0%
POFP [kg NMVOC]	Lotusan®	1.5E+00	58.9%	8.3E-02	3.4%	1.9E-02	0.8%	2.1E-01	8.6%	6.8E-01	27.3%	2.5E-02	1.0%	2.5E+00	100.0%
	Jumbosil®	1.9E+00	58.5%	1.2E-01	3.6%	2.7E-02	0.9%	3.1E-01	9.5%	8.5E-01	26.4%	3.6E-02	1.1%	3.2E+00	100.0%
ALOP [m^{2}a]	Lotusan®	1.9E+01	60.0%	8.6E+00	26.7%	5.8E-01	1.8%	7.0E-01	2.2%	2.6E+00	8.2%	4.1E-01	1.3%	3.2E+01	100.0%
	Jumbosil®	1.2E+01	40.6%	1.2E+01	40.6%	8.3E-01	2.7 %	1.0E+00	3.3%	3.3E+00	10.8%	5.9E-01	1.9%	3.1E+01	100.0%
$USEtox_{humantox}$ [CTU]	Lotusan®	5.2E-05	68.7%	4.0E-06	5.4%	6.2E-07	0.8%	3.3E-06	4.4%	1.5E-05	20.0%	5.1E-07	0.7%	7.5E-05	100.0%
	Jumbosil®	4.7E-05	60.8%	5.6E-06	7.3%	8.9E-07	1.2%	4.7E-06	6.2%	1.9E-05	24.6%	0.0E+00	0.0%	7.7E-05	100.0%
$USEtox_{ecotox}$ [CTU]	Lotusan®	1.3E+03	90.5%	1.1E+01	0.7%	2.1E+00	0.1%	2.2E+01	1.5%	9.8E+01	6.8%	4.3E+00	0.3%	1.4E+03	100.0%
	Jumbosil®	1.0E+03	85.2%	1.6E+01	1.3%	3.1E+00	0.3%	3.1E+01	2.6%	1.2E+02	10.2%	6.2E+00	0.5%	1.2E+03	100.0%
PMFP [PM10e]	Lotusan®	9.0E-01	70.7%	3.9E-02	3.0%	8.6E-03	0.7%	7.0E-02	5.5%	2.5E-01	19.6%	6.6E-03	0.5%	1.3E+00	100.0%
	Jumbosil®	9.5E-01	65.9%	5.4E-02	3.8%	1.3E-02	0.9%	1.0E-01	7.0%	3.1E-01	21.8%	9.3E-03	0.7%	1.4E+00	100.0%

[a]All values are related to the functional unit. It has to be noted that values may contain impact indicator-specific rounding differences.

Table S4: Detailed analysis for the contributions arising from the bill of formulation components[a].

Impact category	Façade paint	Water	Pigment (TiO_2)	Polymer dispersion (butyl acrylate)	Polymer dispersion (methyl methacrylate)	Polymer dispersion (styrene)	Polymer dispersion (water)	Filler (rock flour)	Chemicals, inorganic	Chemicals, organic	Fibres (cellulose)	Silicone resin	Hydrophobizing agent	Transport services	Sum
$CED_{non\text{-}renewable}$	Lotusan®	0.01%	68.47%	3.96%	5.06%	6.65%	<0.01%	0.04%	0.76%	1.39%	0.15%	9.52%	2.48%	1.50%	100%
	Jumbosil®	0.01%	38.01%	13.78%	18.22%	22.43%	0.01%	0.04%	2.14%	3.56%	---	---	---	1.80%	100%
GWP_{100a}	Lotusan®	0.01%	72.24%	2.94%	4.70%	7.05%	<0.01%	0.05%	1.17%	0.59%	0.10%	7.05%	1.76%	2.35%	100%
	Jumbosil®	<0.01%	36.28%	11.30%	18.44%	25.58%	<0.01%	0.06%	2.38%	2.38%	---	---	---	3.57%	100%
WDP	Lotusan®	1.49%	93.62%	0.41%	0.05%	0.52%	0.26%	0.31%	0.36%	0.10%	0.05%	2.11%	0.53%	0.18%	100%
	Jumbosil®	1.78%	86.16%	2.78%	0.35%	3.58%	1.80%	0.73%	1.86%	0.49%	---	---%	---	0.47%	100%
TAP_{100a}	Lotusan®	<0.01%	83.75%	1.92%	3.20%	2.57%	<0.01%	0.12%	0.93%	0.51%	0.08%	4.75%	1.19%	0.97%	100%
	Jumbosil®	<0.01%	60.18%	8.32%	13.86%	11.11%	<0.01%	0.18%	3.04%	1.68%	---	---	---	1.62%	100%
FEP	Lotusan®	0.01%	84.96%	1.77%	0.67%	2.49%	<0.01%	0.02%	1.02%	0.49%	0.14%	6.74%	1.68%	0.01%	100%
	Jumbosil®	0.01%	65.40%	10.07%	3.81%	14.18%	0.01%	0.03%	4.36%	2.10%	---	---	---	0.02%	100%
MEP	Lotusan®	<0.01%	73.86%	1.01%	5.99%	1.60%	<0.01%	0.13%	0.66%	0.33%	0.17%	5.57%	1.39%	9.29%	100%
	Jumbosil®	<0.01%	43.50%	4.41%	26.10%	6.96%	<0.01%	0.20%	2.18%	1.08%	---	---	---	15.56%	100%
POFP	Lotusan®	<0.01%	76.43%	2.49%	4.93%	4.15%	<0.01%	0.29%	0.78%	0.86%	0.14%	5.91%	1.48%	2.55%	100%
	Jumbosil®	<0.01%	42.74%	10.24%	20.38%	17.06%	<0.01%	0.41%	2.44%	2.68%	---	---	---	4.04%	100%
ALOP	Lotusan®	0.01%	63.06%	1.50%	0.03%	1.74%	<0.01%	0.03%	1.07%	0.18%	7.08%	21.02%	4.28%	<0.01%	100%
	Jumbosil®	0.01%	66.94%	11.74%	0.20%	13.63%	0.01%	0.09%	6.34%	1.04%	---	---	---	0.01%	100%
$USEtox_{humantox}$	Lotusan®	0.01%	89.43%	1.29%	0.44%	2.04%	<0.01%	0.02%	0.91%	0.32%	0.19%	3.92%	0.98%	0.45%	100 %
	Jumbosil®	0.01%	71.29%	7.60%	2.59%	11.99%	0.01%	0.05%	4.03%	1.41%	---	---	---	1.03%	100%
$USEtox_{ecotox}$	Lotusan®	<0.01%	97.46%	0.16%	0.11%	0.99%	<0.01%	0.00%	0.33%	0.11%	0.03%	0.49%	0.14%	0.16%	100%
	Jumbosil®	<0.01%	89.42%	1.21%	0.60%	6.34%	<0.01%	0.01%	1.51%	0.60%	---	---	---	0.30%	100%
PMFP	Lotusan®	<0.01%	83.59%	1.84%	2.59%	2.35%	<0.01%	0.59%	0.85%	0.54%	0.11%	5.15%	1.29%	1.10%	100%
	Jumbosil®	<0.01%	57.34%	9.30%	13.04%	11.86%	<0.01%	1.04%	3.23%	2.05%	---	---	---	2.14%	100%

[a]Results are given for the provision of raw materials required for the production of 1 kg of each façade paint.

References

[1] ecoinvent Centre Ed. ecoinvent data V3.01. Swiss Centre for Life Cycle Inventories, 2013. www.ecoinvent.org (accessed July 16, 2015).

[2] Hischier, R.; Weidema, B., Eds. Implementation of Life Cycle Impact Assessment Methods. Data V2.2 2010, *ecoinvent report No. 3*, St. Gallen, Switzerland.

[3] Change, Climate. "Intergovernmental Panel on Climate Change." World Meteorological Organization, 2007.
